# Morphology, photosynthetic physiology and biochemistry of nine herbaceous plants under water stress

**DOI:** 10.3389/fpls.2023.1147208

**Published:** 2023-03-30

**Authors:** Qiaoyu Luo, Huichun Xie, Zhi Chen, Yonggui Ma, Haohong Yang, Bing Yang, Yushou Ma

**Affiliations:** ^1^ School of Life Sciences, Qinghai Normal University, Xining, China; ^2^ Qinghai Provincial Key Laboratory of Medicinal Plant and Animal Resources of Qinghai-Tibet Plateau, Qinghai Normal University, Xining, China; ^3^ Academy of Plateau Science and Sustainability, Qinghai Normal University, Xining, China; ^4^ College of Agriculture and Animal Husbandry, Qinghai University, Xining, China; ^5^ Sichuan Academy of Giant Panda, Chengdu, China

**Keywords:** plant physiology, drought, waterlogging, morphology, chlorophyll content, lipid oxidation, malodehyde, osmoprotectant

## Abstract

Global climate warming and shifts in rainfall patterns are expected to trigger increases in the frequency and magnitude of drought and/or waterlogging stress in plants. To cope with water stress, plants develop diverse tactics. However, the adoption capability and mechanism vary depending upon the plant species identity as well as stress duration and intensity. The objectives of this study were to evaluate the species-dependent responses of alpine herbaceous species to water stress. Nine herbaceous species were subjected to different water stresses (including moderate drought and moderate waterlogging) in pot culture using a randomized complete block design with three replications for each treatment. We hypothesized that water stress would negatively impact plant growth and metabolism. We found considerable interspecies differences in morphological, physiological, and biochemical responses when plants were exposed to the same water regime. In addition, we observed pronounced interactive effects of water regime and plant species identity on plant height, root length, root/shoot ratio, biomass, and contents of chlorophyll a, chlorophyll b, chlorophyll (a+b), carotenoids, malondialdehyde, soluble sugar, betaine, soluble protein and proline, implying that plants respond to water regime differently. Our findings may cast new light on the ecological restoration of grasslands and wetlands in the Qinghai-Tibetan Plateau by helping to select stress-tolerant plant species.

## 1 Introduction

Grasslands and wetlands play a critical role in water and soil conservation, flood storage, maintaining productivity, cycling and storing carbon and sustaining biodiversity (Wang et al., 2021; Wang Y. et al., 2022). The Qinghai–Tibetan Plateau is dominated by alpine grassland, which accounts for more than 60% of the total area of the Qinghai–Tibetan Plateau (Li et al., 2023) and is important for its contributions to the aforementioned processes (Wang Y. et al., 2022). However, approximately 70% of alpine grassland has been degraded in recent decades (Peng et al., 2019). The degradation of grasslands has resulted in soil erosion (Liu et al., 2018; Li et al., 2019; Dai et al., 2021; Li et al., 2023) and biodiversity loss (Wang et al., 2009; Yu et al., 2022).

Ongoing climate change is expected to induce extremes in drought and flooding (Bailey-Serres et al., 2012), particularly in the Qinghai-Tibetan Plateau region, and thus, the plants here are likely to be subjected to frequent drought and waterlogging stress concurrently under global warming (Luo et al., 2022). To reduce and eliminate the deleterious effects of degrading grasslands on ecosystem functioning, it is of utmost importance to develop and adopt germplasms that have a better capacity to endure abiotic stress. A comprehensive understanding of the developmental, morphological, and physiological responses of plants to extremes in water availability is a prerequisite to identify high-quality germplasm resources. Waterlogging stress or drought stress, the major environmental stresses that plants encounter during their growth and development stages (Bello et al., 2022), limits plant survival, reproduction and yield (Kar, 2011). Previous studies have demonstrated that water stress induces changes in morphological, physiological, and biochemical plant characteristics to counteract potential harm (Hsiao, 1973; Nautiyal et al., 1994; Fernández et al., 2002). However, the degree of adaptation of plants to water stress may vary considerably among environmental conditions (including soil fertility and climate) and within species. Moreover, there are complex interactions across plant species, water stress intensity and duration and environmental factors. Therefore, the response and adaptation mechanisms of plants to water stress remain unclear. However, the response and adaptation mechanisms of plants are very important for breeding water-tolerant varieties (Wu et al., 2022).

Plant height is an important trait used to indicate competition capability, plant growth and production (Jiang et al., 2020). Plant roots are also an important component of the drought stress response (Guo et al., 2020). The root/shoot ratio is an alternative measurement method and is frequently employed to capture the biomass allocation of plants (Poorter et al., 2012) or reflect the differential investment of photosynthesis between the aboveground and belowground organs (Titlyanova et al., 1999). An increased root/shoot ratio suggests more investment of photosynthesis into belowground parts. According to the “optimal partitioning theory” (Gedroc et al., 1996), plants preferentially allocate biomass and nonstructural carbohydrates to acquire the resource that most limits their growth (Kobe et al., 2010). For example, drought increased the root/shoot ratio in rice (Xu et al., 2015) and Sage (Caser et al., 2017), and waterlogging reduced the root/shoot ratio in winter wheat (Shao et al., 2013) and maize (Herzog et al., 2016). Photosynthetic pigments play a role in the absorption, transmission and transformation of light energy during photosynthesis (Arjenaki et al., 2012; Reinbothe et al., 2010). Chlorophyll a mainly converts the collected light energy into chemical energy for photochemistry, while chlorophyll b mainly collects light energy. Although both chlorophyll a and chlorophyll b can receive and transmit light energy, only part of chlorophyll a can act as the central pigment of photosynthetic reactions (Reddy et al., 2004). Chlorophyll concentration is known as an indicator for the evaluation of photosynthesis (Zobayed et al., 2005), and its decline has been considered a nonstomatal limiting factor and a kind of protection mechanism for photosynthetic structures under abiotic stress (Du et al., 2012; Jumrani and Bhatia, 2019; Bhusal et al., 2020a). The contents of chlorophyll a and chlorophyll b directly affect the photosynthesis and growth status of plants to a certain extent (Paknejad et al., 2006). Carotenoids overcome the effects of stress on plant growth by helping maintain photosynthesis and reducing the degree of membrane oxidative damage (Paknejad et al., 2006). Photosynthetic activity is inhibited in plant tissues due to an imbalance between light capture and its utilization under drought stress (Foyer and Noctor, 2000). Malondialdehyde (MDA) can well indicate the degree of membrane lipid peroxidation (Rui et al., 2013; Zhou et al., 2022), and its content has been used to reflect the response of plants to stress (Gill and Tuteja, 2010; Wang X. et al., 2022; Zhou et al., 2022). Osmotic adjustment substances such as proline (Pro), soluble sugars (SS), and soluble protein (SP) can effectively reduce the water potential of plant cells under drought conditions and prevent cell dehydration to ensure normal plant growth (Ozturk et al., 2021; Zhou et al., 2022). For instance, Pro accumulates as an adaptive response under stress conditions (Maggio et al., 2002; Zulfiqar and Ashraf, 2022) to protect plants from the deleterious impact of water deficiency-mediated oxidative stress by increasing ROS quenching efficiency *via* different mechanisms, including maintaining GSH/GSSG balance. In addition, its accumulation aids in retaining membrane integrity by decreasing lipid oxidation by guarding the cellular redox potential and scavenging free radicals (Shinde et al., 2016; Nadeem et al., 2019). Finally, inhibited activities of Pro dehydrogenase and Pro oxidase by water stress slow the incorporation of Pro into protein (Kumar et al., 2020).

In the present study, we compared the performance of nine herbaceous species in different soil water conditions stimulating global climate change. As environmental stress induces multiple responses in plants, from subcellular to structural levels, major morphological, physiological, and biochemical parameters were assessed. We aimed to answer the following questions: (1) Is plant species identity an important factor determining the morphological, physiological and biochemical responses to environmental change? (2) Does environmental stress change the inherent differences in growth and metabolism across plant species? We hypothesized that water stress would impact plant growth and metabolism. We also expected that the effect of moderate water stress would be less pronounced than that of plant species identity.

## 2 Materials and methods

### 2.1 Substrate

To exclude the potential effects of soil texture, fertility and soil biota on plant growth and metabolism, all soil used in the present study was of the same source. The soil was collected from an alpine meadow in Dawu Town, Maqing County, Golog Tibetan Ethnic Minority Autonomous Prefecture, Qinghai Province, while the sands were purchased from a building materials market nearby. As previously described (Luo et al., 2022), the soil of the alpine meadow was classified as Mat Cry-gelic Cambisol, and its chemical properties are as follows: soil organic matter 14.53 mg/g, total nitrogen 3.12 mg/g, total phosphorus 0.26 mg/g, total potassium 19.58 mg/g, pH 7.63 (water/soil at 1:1 weight/volume) and CEC 225.52 μS/cm (water/soil at 5:1 weight/volume).

### 2.2 Plant material

In the present study, nine herbaceous species including *Deschampsia caespitosa*, *Poa crymophila* Keng, *Poa pratensis* L. cv. Qinghai, *Festuca sinensis* Keng ex S. L. Lu, *Puccinellia tenuiflora* (Griseb.) Scribn. et Merr. cv. Tongde, *Elymus nutans* Griseb., *Kobresia tibetica*, *Blysmus sinocompressus* Tang et Wang, and *Carex moorcroftii* Falc. Ex Boott were tested. All selected plants were supplied with root nutrients. *D. caespitosa*, *P. crymophila*, *P. pratensis*, *F. sinensis*, *P. tenuiflora*, and *E. nutans* were obtained from the seed breeding fields of the Grassland Research Institute, Academy of Animal Husbandry and Veterinary Sciences, Qinghai University, which is located in Dawu Town, Maqin County, Golog Tibetan Autonomous Prefecture, Qinghai Province, whereas *K. tibetica*, *B. sinocompressus* and *C. moorcroftii* were collected from the nearby alpine meadow of the seed breeding field. In early May 2018, the plants with rootstalk were dug up, and then litter were removed. The plants were carefully divided into small clusters with approximately the same amount of root and aboveground biomass and kept at moderate moisture for later use.

### 2.3 Experimental setup

The study was conducted at the Chengbei Campus of Qinghai Normal University (36°44′N, 101°44′E), Xining city, Qinghai Province, China. In May 2018, each cluster of the nine plant species was transplanted into a pot (20 cm in diameter, 25 cm in height) containing 3.0 kg of a mixture of alpine meadow soil and sand (sand/soil at 1:1 weight/volume). Seedlings were kept at 10 individual plants per pot after seedlings survived. During this period, the plants were kept in the greenhouse and watered when needed. To ensure that all replicates of each treatment had similarly healthy and representative individuals, only those growing well were kept for later use. In July 2018, water stress treatment was carried out using a completely randomized design.

During water treatment, the canopy was erected in situ. Both sides of the canopy were ventilated, which did not affect the temperature and humidity. Thereafter, the daytime and nighttime temperatures of the greenhouse were continuously managed to mimic environmental field conditions. During the experiment, the daytime temperature was (20 ± 2)°C, and the nighttime temperature was (5 ± 2)°C. The day length of the interior climate was that of the outside environmental conditions since the transparent, clear glass chamber structure was completely exposed to the outside environment. The structure was also equipped with automatic vents and fans. Air temperature and relative humidity inside the canopy were monitored with a portable meteorological meter (Holder HED-SQ, China).

Three water treatments were set up as follows: moderate waterlogging (only the root and neck of the plant was flooded, that is, the depth of the water was approximately 3 cm, MW), normal plant water requirement (70%-80% of field water capacity, control (CK), and moderate drought (30%-40% of field water capacity, MD). There were 10 replicates for each treatment. Soil moisture was monitored by the combined weighting method and soil moisture sensor (ProCheck, USA), and the lost water was replenished every two days to ensure that the plants were living under the given soil moisture. Watering was performed between 18:00~19:00, and a plant-free pot was set as a control to estimate water loss due to evaporation. The water stress treatments lasted for 35 days.

### 2.4 Sampling and assaying

#### 2.4.1 Determination of biomass

Sampling was conducted on the 36th day after treatment. Five individual plants were randomly selected from each pot to measure height and root length using a measuring tape. Then, three pots of each treatment were randomly selected and harvested manually, and the biomass of aboveground parts, including stems and leaves, and underground root biomass were collected separately. The plants were cut with scissors at 5 cm above the soil. Stems and leaves were collected and put into a kraft bag, and roots were removed carefully and washed. All the collected plant materials were desiccated at 105°C and oven-dried at 80°C to determine the dry weight. The plants of the remaining seven pots were collected, snap-frozen and stored at –80°C for biochemical assays (contents of photosynthetic pigment, MDA and osmoprotectant). The root/shoot ratio was estimated by dividing the total dry root biomass by the total dry shoot biomass of each pot.

#### 2.4.2 Photosynthetic pigment determination

Chlorophyll was extracted using acetone and anhydrous ethanol. Briefly, approximately 0.1 g of fresh leaves was weighed, cut and put into a calibration test tube. Then, 10 mL of a mixture of 95% ethanol and 80% acetone at a volume ratio of 1:1 was added and incubated in the dark for 48 hours until the green leaves became colorless. A mixture of 95% ethanol and 80% acetone was used as a blank control. The absorbance values were measured at 470 nm, 645 nm and 663 nm by an enzyme-labeled instrument (Bole xMark). Absorbance values were calculated using the following equations:


$$Chlorophyll\;a\;=\;[\left(12.72\;A_{663}-2.59\;A_{645}\right)\times V\times N/W$$

$$Chlorophyll\;b\;=\;[\left(22.88\;A_{645}-4.67\;A_{663}\right)\times V\times N/W$$

Chlorophyll = chlorophyll a + chlorophyll b

$$Carotenoid\;=\;\left(1000\;A_{470}-2.05\;Chlorophyll\;a-114.8\;Chlorophyll\;b\right)/245\times V\times N/W$$

where V represents the volume of the extract, N represents the dilution, and W represents the fresh weight of the sample (g).

#### 2.4.3 MDA assaying

Lipid peroxidation, an indicator of oxidative damage to the cell membranes (Girotti, 1990), was estimated by measuring MDA production (Dhindsa and Matowe, 1981). Briefly, frozen leaf samples (0.5 g) were ground to a powder in a mortar with liquid nitrogen and homogenized with 2 mL phosphate buffer (PBS, pH 7.8). The resulting residue was washed three times with 1 mL PBS each time and pooled into a centrifuge tube. The homogenate was centrifuged at 6000 rpm for 20 min. The supernatant was used to measure MDA. The supernatant (1 mL) was added to 5 mL of 0.5% TCA containing 0.6% thiobarbituric acid (TBA). The solution was boiled for 10 min and then centrifuged at 12000 rpm for 10 min after cooling. The absorbance of the mixture was measured at 450 nm, 532 nm, and 600 nm. The MDA content was estimated according to the following equation:


$$C\left(MDA\right)/\mu mol\cdot L^{-1}=\;\left[6.452\times \left(A_{532}-A_{600}\right)\;-0.559\times A_{450}\right]\;\times V1/\left(W\times V2\right)$$

where V1 represents the total volume of the extract; V2 represents the volume of sample solution during measurement; and W is the fresh weight of the sample (g).

#### 2.4.4 Determination of osmotic adjustment substances

The SS content was determined using the anthrone method (John et al., 1950). The content of SP was determined using the G-250 Coomassie brilliant blue method (Bradford, 1976). A 1 g frozen sample was ground with 1.5 mL 80% ethanol (adding a little quartz sand) in the precooling bowl, and the volume was fixed to 5 mL with 80% ethanol solution. The extract was transferred into the test tube at 80°C for 20 min. Then, the extract was filtered twice through filter paper with activated carbon. The filtrate was placed in the test tube with 0.2 × the weight of zeolite and oscillated for 5 min. The supernatant was centrifuged at 4°C for 10 min at 5000 × g, and Procontent was determined by acid ninhydrin colorimetry (Bates et al., 1973).

### 2.5 Statistical analysis

Before analysis, the normality and variance homogeneity of variables were examined by using the Shapiro−Wilk normality test and Levene’s test, respectively. When the assumption was met, means were compared with two-way ANOVAs, and multiple comparisons were carried out using Tukey’s HSD test. Otherwise, means were compared with the Kruskal−Wallis test, and multiple comparisons were performed using the Wilcox test with the Benjamini method to adjust the *P* values. All statistics were performed using Statistical Product and Service Solutions (SPSS v22.0, IBM Corporation, United States). The figures were produced using OriginPro 2017 (OriginLab Corp, Northampton, United States).

## 3 Results

### 3.1 Morphology and biomass

We found a significant effect of water stress on plant height, root length, total biomass, and root/shoot ratio and distinct interspecies differences in plant height, total biomass, and root/shoot ratio (**Tables 1**, **A1**). Additionally, we also observed significant interactive effects of water stress and species identity on plant height, biomass and root/shoot ratio (**Tables 1**, **A1**).

**Table 1:** Plant height, root length, plant biomass and root/shoot ratio of nine selected herbaceous plant species under different water regimes, including control (CK), moderate drought (MD), and moderate waterlogging (MW).

Index	Water regime	*Deschampsia caespitosa*	*Poa crymophila*	*Poa pratensis*	*Festuca sinensis*	*Puccinellia tenuiflora*	*Elymus nutans*	*Kobresia tibetica*	*Blysmus sinocompressus*	*Carex moorcroftii*
Plant height (cm)	MW	43.09 ± 2.29 ABa	37.67 ± 4.33 ABCb	34.33 ± 2.91 BCa	46.55 ± 1.13 Aa	42.89 ± 3.40 ABa	42.00 ± 4.19 ABa	24.11 ± 0.73 Da	32.22 ± 0.78 Cab	43.89 ± 1.16 Aa
	CK	38.40 ± 0.86 ABab	38.78 ± 0.73 ABab	33.66 ± 3.38 ABCa	41.89 ± 1.42 Aa	33.89 ± 7.53 ABCa	37.22 ± 1.06 ABa	25.89 ± 3.31 Ca	36.89 ± 2.63 ABa	29.33 ± 0.38 BCc
	MD	32.12 ± 4.64 ABCb	41.33 ± 5.77 Aa	36.33 ± 1.00 ABa	33.45 ± 3.01 ABCb	41.33 ± 1.00 Aa	26.89 ± 1.22 Cb	30.50 ± 0.87 BCa	30.50 ± 1.25 BCb	38.66 ± 1.54 ABb
Root length (cm)	MW	16.77 ± 0.41 ABCa	15.78 ± 2.44 ABCa	10.78 ± 1.46 Ca	18.72 ± 1.16 ABa	12.44 ± 1.49 BCa	13.52 ± 0.75 ABCb	18.56 ± 3.51 ABa	12.72 ± 1.45 BCa	19.83 ± 2.77 Aa
	CK	18.29 ± 1.25 ABa	16.55 ± 1.18 ABCa	10.89 ± 2.02 Da	12.73 ± 1.83 CDa	10.28 ± 0.94 Da	17.22 ± 0.87ABCa	14.00 ± 1.76 BCDa	12.56 ± 0.11 CDa	19.50 ± 1.83 Aa
	MD	16.73 ± 1.61 ABa	18.00 ± 0.51 ABa	16.00 ± 1.07ABCa	18.13 ± 3.09 ABa	12.11 ± 0.73 Ca	16.78 ± 0.59 ABa	17.73 ± 1.19 ABa	14.17 ± 1.44 BCa	19.50 ± 0.48 Aa
Total biomass (g/pot)	MW	5.74 ± 0.32 Cc	4.44 ± 0.25 Cc	8.17 ± 0.68 Ba	4.86 ± 0.38 Cb	8.80 ± 0.96 ABa	4.26 ± 0.32 Cc	8.07 ± 0.21 Ba	9.18 ± 0.44 Ba	12.00 ± 0.63 Aa
	CK	8.47 ± 0.23 ABa	6.43 ± 1.66 BCa	5.41 ± 0.57 Cb	6.72 ± 0.21BCa	7.71 ± 1.08 ABCa	9.78 ± 0.36 Aa	7.42 ± 0.25 ABCa	6.41 ± 0.72 BCb	8.81 ± 0.87 ABb
	MD	6.87 ± 0.18 ABb	8.18 ± 0.52 Ab	5.75 ± 0.47 BCb	4.13 ± 0.20 Db	6.10 ± 0.38 Ba	7.71 ± 0.39 Ab	6.09 ± 0.21Bb	4.70 ± 0.67 CDb	7.08 ± 0.57 ABb
Root/shoot ratio	MW	0.60 ± 0.06 Bb	1.45 ± 0.19 Aa	0.75 ± 0.05 Ba	0.54 ± 0.12 Bb	0.53 ± 0.06 Bab	0.56 ± 0.10 Bc	0.63 ± 0.05 Bb	1.41 ± 0.05 Ab	0.58 ± 0.05 Ba
	CK	0.60 ± 0.03 Db	1.06 ± 0.07 Aa	0.65 ± 0.05 CDa	0.56 ± 0.09 Db	0.35 ± 0.07 Eb	0.98 ± 0.06 ABb	0.99 ± 0.09 ABa	1.12 ± 0.06 Ab	0.82 ± 0.06 BCa
	MD	1.60 ± 0.13 Ba	0.87 ± 0.18 Db	0.72 ± 0.08 Da	1.31 ± 0.07 BCa	0.73 ± 0.05 Da	1.34 ± 0.09 BCa	1.08 ± 0.10 CDa	2.94 ± 0.18 Aa	0.81 ± 0.09 Da

Different lowercase letters within the same row indicate that there are significant differences between water regimes for the same plant species at α=0.05; Different capital letters within the same column indicate that there are significant differences between plant species under the same water condition at α=0.05. Data are expressed as means ± SEM (n=3).

In comparison with CK, moderate waterlogging significantly increased the plant height of *C. moorcroftii*, whereas moderate drought significantly decreased the plant heights of *C. moorcroftii*, *E. nutans* and *F. sinensis*. Additionally, the heights of *C. moorcroftii*, *D. caespitosa*, and *F. sinensis* under moderate waterlogging were significantly higher than those under moderate drought. The opposite was the case for *E. nutans* and *P. crymophila*.

In comparison with CK, the root length of nine selected plant species under moderate drought did not significantly change. In contrast, the response of root length to moderate waterlogging varied greatly depending on plant species identity. In comparison with CK, moderate waterlogging significantly decreased the root length of *E. nutans*, whereas moderate drought exerted no significant effect on the root length of *E. nutans*. However, the root length of *E. nutans* under moderate waterlogging was significantly shorter than that under moderate drought.

In comparison with CK, both moderate waterlogging and moderate drought significantly decreased the biomasses of *D. caespitosa*, *E. nutans*, *F. sinensis* and *P. crymophila*. In contrast, moderate waterlogging significantly increased the biomasses of *B. sinocompressus* and *C. moorcroftii* in comparison with CK. In addition, the biomasses of *D. caespitosa*, *E. nutans* and *P. crymophila* under moderate waterlogging were significantly less than those under moderate drought. The opposite pattern was observed for *B. sinocompressus*, *C. moorcroftii*, *K. tibetica and P. pratensis*. However, only the biomasses of *D. caespitosa*, *E. nutans* and *P. crymophila* showed significant differences across water regimes.

In comparison with CK, moderate waterlogging significantly decreased the root/shoot ratios of *E. nutans* and *K. tibetica*, and moderate drought significantly increased the root/shoot ratios of *B. sinocompressus*, *D. caespitosa*, *E. nutans*, *F. sinensis and P. tenuiflora*, while significantly decreasing that of *P. crymophila*. However, only the root/shoot ratio of *E. nutans* showed significant differences across water regimes.

### 3.2 Photosynthetic pigments

Water regime, species identity and their interactive effect significantly affected the contents of chlorophyll a, chlorophyll b, chlorophyll (a+b), and carotenoids (**Tables 2**, **3**), suggesting that photosynthetic pigment contents in plants respond differently to water stress.

**Table 2:** Chlorophyll a, chlorophyll b, chlorophyll (a+b) and carotenoid contents of nine herbaceous plant species under different water regimes, including control (CK), moderate drought (MD), and moderate waterlogging (MW).

Index	Water regime	*Deschampsia caespitosa*	*Poa crymophila*	*Poa pratensis*	*Festuca sinensis*	*Puccinellia tenuiflora*	*Elymus nutans*	*Kobresia tibetica*	*Blysmus sinocompressus*	*Carex moorcroftii*
chl.a (mg/g FW)	MW	1.28 ± 0.11Ca	1.41 ± 0.01Ca	0.63 ± 0.07Da	0.74 ± 0.20Db	0.47 ± 0.05Da	0.57 ± 0.08Da	2.32 ± 0.09ABa	2.15 ± 0.23Ba	2.57 ± 0.08Aa
	CK	1.42 ± 0.08BCa	1.07 ± 0.02Dc	0.73 ± 0.10Ea	1.34 ± 0.09BCa	0.50 ± 0.07Ea	0.73 ± 0.06Ea	1.57 ± 0.08ABb	1.32 ± 0.09Cb	1.73 ± 0.06Ab
	MD	0.90 ± 0.04Bb	1.25 ± 0.07Ab	0.84 ± 0.02Ba	0.74 ± 0.10Bb	0.32 ± 0.09Ca	0.71 ± 0.07Ba	0.73 ± 0.06Bc	0.41 ± 0.05Cc	0.81 ± 0.12Bc
chl.b (mg/g FW)	MW	0.55 ± 0.04BCb	0.50 ± 0.01Ca	0.22 ± 0.02DEa	0.27 ± 0.06Da	0.16 ± 0.02Ea	0.21 ± 0.03DEa	0.85 ± 0.03Aa	0.62 ± 0.01Ba	0.49 ± 0.04Ca
	CK	0.63 ± 0.07Aa	0.42 ± 0.02Bb	0.25 ± 0.03Ca	0.24 ± 0.08Ca	0.24 ± 0.08Ca	0.25 ± 0.02Ca	0.59 ± 0.04Ab	0.27 ± 0.03Cb	0.29 ± 0.02BCb
	MD	0.33 ± 0.01Bc	0.44 ± 0.02Aab	0.30 ± 0.01BCa	0.26 ± 0.04BCDa	0.13 ± 0.02Ea	0.20 ± 0.05DEa	0.25 ± 0.02BCDc	0.13 ± 0.02Ec	0.23 ± 0.03CDc
Chl (mg/g FW)	MW	1.83 ± 0.12Ba	1.91 ± 0.23Ba	0.85 ± 0.09Ca	1.00 ± 0.27Ca	0.63 ± 0.07Ca	0.79 ± 0.10Ca	3.17 ± 0.10Aa	2.76 ± 0.23Aa	3.06 ± 0.08Aa
	CK	2.05 ± 0.09Aa	1.49 ± 0.03Bc	0.98 ± 0.13Ca	1.57 ± 0.01Ba	0.74 ± 0.16Ca	0.98 ± 0.08Ca	2.16 ± 0.08Ab	1.59 ± 0.07Bb	2.01 ± 0.04Ab
	WD	1.23 ± 0.06Bb	1.69 ± 0.08Ab	1.14 ± 0.03BCa	1.00 ± 0.14BCa	0.45 ± 0.11Da	0.91 ± 0.11Ca	0.98 ± 0.08BCc	0.53 ± 0.06Dc	1.04 ± 0.13BCc
Cx.c (mg/g FW)	MW	0.39 ± 0.04Ba	0.43 ± 0.02Ba	0.18 ± 0.01CDb	0.23 ± 0.07Ca	0.16 ± 0.01CDa	0.17 ± 0.02CDa	0.12 ± 0.02Db	0.58 ± 0.03Aa	0.38 ± 0.03Ba
	CK	0.39 ± 0.03ABa	0.32 ± 0.04ABCb	0.23 ± 0.02Cab	0.21 ± 0.08Ca	0.21 ± 0.08Ca	0.21 ± 0.01Ca	0.17 ± 0.02Cab	0.45 ± 0.03Ab	0.29 ± 0.03BCab
	MD	0.26 ± 0.01Bb	0.42 ± 0.05Aa	0.26 ± 0.01Ba	0.23 ± 0.03BCa	0.12 ± 0.02Da	0.21 ± 0.02BCa	0.21 ± 0.01BCa	0.12 ± 0.02Dc	0.18 ± 0.04CDb

Different lowercase letters within the same row indicate that there are significant differences between water regimes for the same plant species at α=0.05; Different capital letters within the same column indicate that there are significant differences between plant species under the same water condition at α=0.05.

**Table 3:** Results of two-way ANOVAs examining the major and interactive effects of water regime and plant species identity on the contents of photosynthetic pigments.

Source of variation	*df*	Chlorophyll a		Chlorophyll b		Chlorophyll (a+b)		Carotenoid	
		F	*P*	F	*P*	F	*P*	F	*P*
Water regime (W)	2	60.821	<0.001	42.405	<0.001	68.274	<0.001	21.619	<0.001
Species identity (S)	8	97.901	<0.001	55.472	<0.001	115.663	<0.001	10.630	<0.001
Interaction (W×S)	16	22.358	<0.001	12.941	<0.001	24.868	<0.001	8.000	<0.001

In comparison with CK, the content of chlorophyll a in leaves of *B. sinocompressus*, *C. moorcroftii* and *K. tibetica* significantly increased under moderate waterlogging but significantly decreased under moderate drought. In contrast, the content of chlorophyll a in leaves of *P. crymophila* significantly increased under water stress, whereas those in leaves of *F. sinensis* significantly decreased. Finally, the chlorophyll a content in the leaves of *D. caespitosa* significantly decreased under moderate drought compared to that of CK. Overall, we observed significant differences in chlorophyll a content in leaves of *B. sinocompressus*, *C. moorcroftii*, *K. tibetica* and *P. crymophila* across water regimes.

In comparison with CK, the content of chlorophyll b in leaves of *B. sinocompressus*, *C. moorcroftii* and *K. tibetica* significantly increased under moderate waterlogging, whereas the opposite held true for those in leaves of *B. sinocompressus*, *C. moorcroftii* and *K. tibetica* under moderate drought. Additionally, the chlorophyll b content in the leaves of *D. caespitosa* significantly decreased under both moderate waterlogging and moderate drought. Finally, the content of chlorophyll b in leaves of *P. crymophila* significantly decreased under moderate waterlogging in comparison with those of CK. Overall, we observed significant differences in the content of chlorophyll b in leaves of *B. sinocompressus*, *C. moorcroftii*, *K. tibetica* and *D. caespitosa* across water regimes.

Moderate waterlogging significantly increased the chlorophyll content in the leaves of *B. sinocompressus*, *C. moorcroftii* and *K. tibetica* in comparison with CK. In contrast,moderate drought significantly decreased the chlorophyll content in the leaves of *B. sinocompressus*, *C. moorcroftii* and *K. tibetica*. However, both moderate waterlogging and moderate drought significantly increased the chlorophyll content in leaves of *P. crymophila*. Finally, chlorophyll content in leaves of *P. crymophila* significantly decreased under moderate drought compared to that of CK. Overall, we observed significant differences in chlorophyll content in leaves of *B. sinocompressus*, *C. moorcroftii*, *K. tibetica* and *P. crymophila* across water regimes. The carotenoid content in leaves of *B. sinocompressus* and *D. caespitosa* significantly increased under moderate waterlogging in comparison with CK. In contrast, the carotenoid contents in leaves of *B. sinocompressus* and *D. caespitosa* significantly decreased under moderate drought in comparison with CK. Additionally, the carotenoid content in leaves of *P. crymophila* significantly increased under moderate drought compared to that of CK. Overall, we only observed significant differences in carotenoid content in leaves of *B. sinocompressus* across water regimes.

### 3.3 Lipid peroxidation

**Figure 1:**
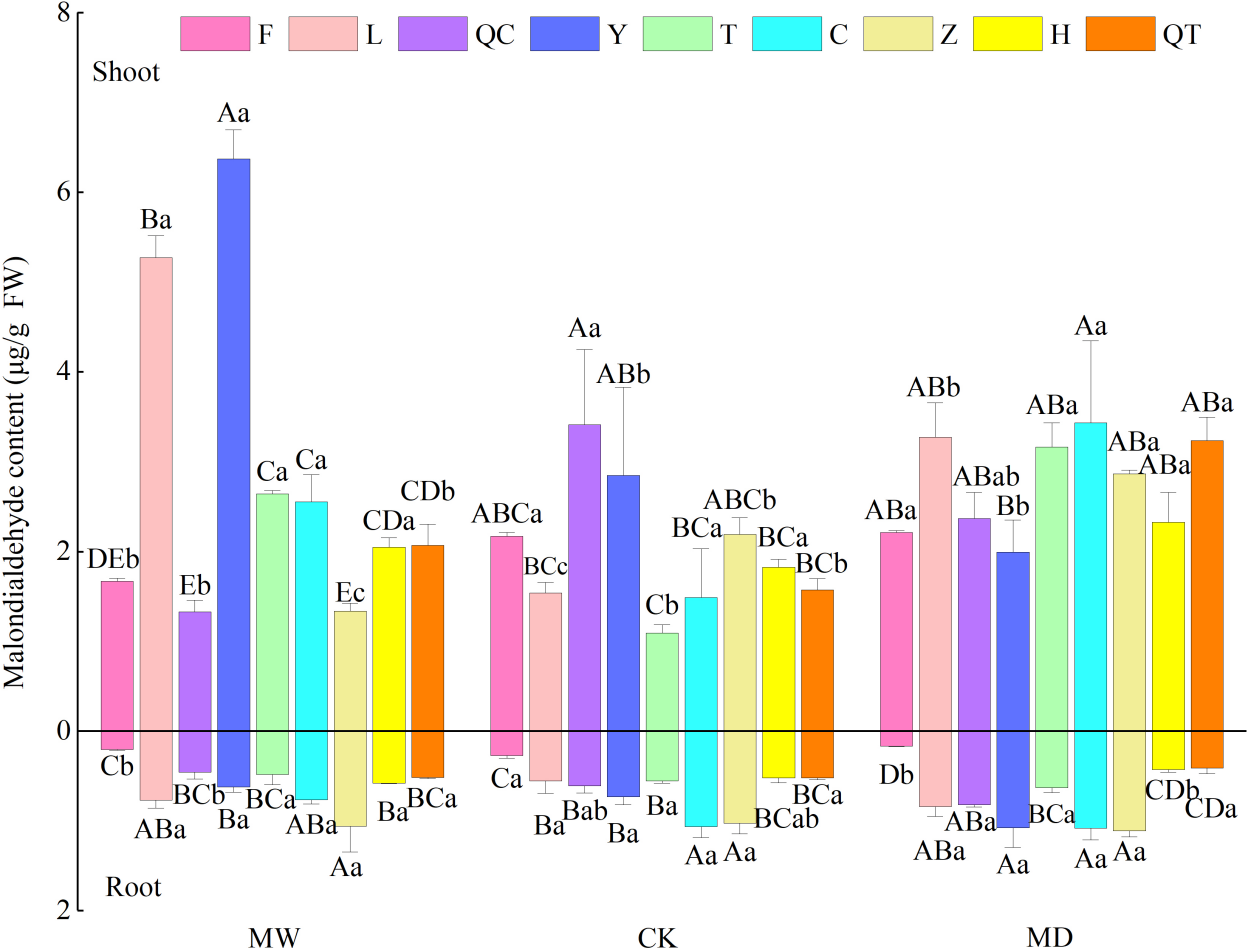
Malonaldehyde contents in leaves of nine selected plant species under different water regimes, including control (CK), moderate drought (MD) and moderate waterlogging (MW). Plant species include *Deschampsia caespitosa* (F), *Poa crymophila* Keng (L), *Poa pratensis* L. cv. Qinghai (QC), *Festuca sinensis* Keng ex S. L. Lu (Y), *Puccinellia tenuiflora* (Griseb.) Scribn.et Merr.cv. Tongde (T), *Elymus nutans* Griseb. (C), *Kobresia tibetica* (Z), *Blysmus sinocompressus* Tang et Wang (H), and *Carex moorcroftii* Falc. Ex Boott (QT). Different lowercase letters indicate that there are significant differences between water regimes for the same plant species at *P*<0.05, different capital letters indicate that there are significant differences between plant species under the same water condition at *P*<0.05. Bars represent the standard error (n = 3).

MDA content in both the shoots and roots of the nine selected plants was changed by the water regime. In addition, MDA content showed a remarkable interspecies difference. Furthermore, we observed significant interactive effects of plant species identity and water regime on MDA contents in shoots of the nine selected plants (**Table A2**; **Figure 1**).

### 3.4 Osmoprotective compounds

**Figure 2:**
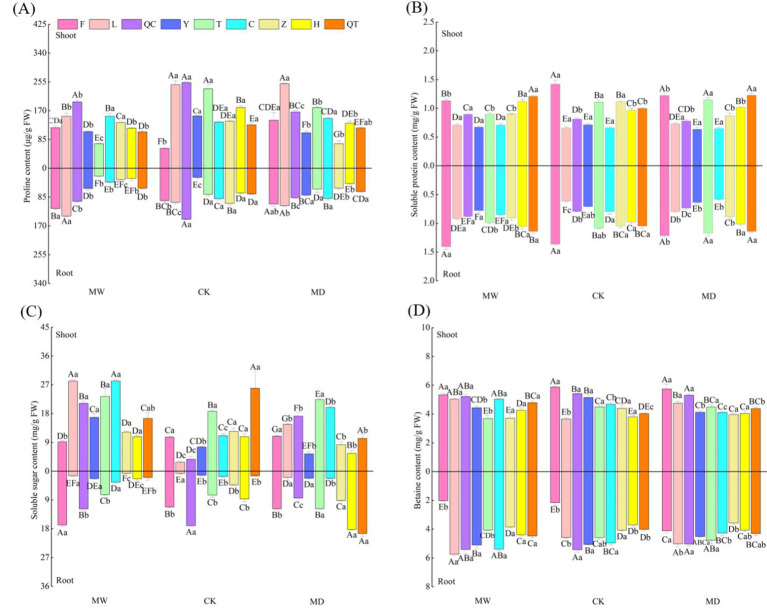
Proline **(A)**, soluble protein **(B)**, soluble sugar **(C)** and betaine **(D)** contents in shoots of nine selected plant species under different water regimes, including control (CK), moderate drought (MD) and moderate waterlogging (MW). Plant species include *Deschampsia caespitosa*(F), *Poa crymophila* Keng (L), *Poa pratensis* L. cv. Qinghai (QC), *Festuca sinensis* Keng ex S. L. Lu (Y), *Puccinellia tenuiflora* (Griseb.) Scribn.et Merr.cv. Tongde (T)>, *Elymus nutans* Griseb. (C), *Kobresia tibetica* (Z), *Blysmus sinocompressus* Tang et Wang (H), and *Carex moorcroftii* Falc. Ex Boott (QT). Different lowercase letters within the same row indicate that there are significant differences between water regimes for the same plant species at *P*<0.05 and different capital letters within the same column indicate that there are significant differences between plant species under the same water condition at *P*<0.05. Bars represent the standard error (n = 3).

The water regime exerted significant effects on the contents of soluble sugar, betaine, soluble protein and proline in both the shoots and roots of the nine selected plants. Plant species identity exerted significant effects on the contents of soluble sugar and proline in the shoots of selected plants as well as on the contents of soluble sugar, betaine, soluble protein and proline in the roots of selected plants (**Table A3**; **Figure 2**). Additionally, the interactive effects of water regime and plant species identity significantly affected the contents of soluble sugar, betaine, soluble protein and proline in plant shoots and roots, suggesting that osmoprotective compound contents in plants respond differently to water stress (**Table A3**; **Figure 2**).

## 4 Discussion

### 4.1 Morphology and biomass

In the present study, water stress including drought and waterlogging, evidently decreased the biomasses of *D. caespitosa*, *E. nutans* and *P. crymophila*, but did not significantly change the biomass of *P. tenuiflora.* However, moderate waterlogging significantly increased the biomasses of *B. sinocompressus* and *C. moorcroftii* (**Table 2**). Our results are in agreement with previous studies that demonstrated that drought stress inhibits plant growth and biomass accumulation (*e.g.*, Loggini et al., 1999; Lenhart et al., 2015; Caser et al., 2019; El-Beltagi et al., 2020; Moreno-Galván et al., 2020; Wang et al., 2021), whereas waterlogging decreases (de San Celedonio et al., 2014; Imaz et al., 2015; Doupis et al., 2017) or increases plant biomass (Rubio et al., 1995). The likely reason why does plant increase biomass under waterlogging is that this plant has a tight regulation of water and carbon relations under severe soil-oxygen deficiency (Insausti et al., 2001). In light of the stability of biomass, we argue that *P. tenuiflora* rather than *D. caespitosa* is a promising species in grassland restoration in the Qinghai-Tibetan Plateau.

In our study, the responses of plants to drought and waterlogging were dependent on plant species identity. Similar results have been reported elsewhere (Reents et al., 2021). In addition, *E. nutans* showed a marked increase in root/shoot ratio when exposed to drought and pronounced lower in root/shoot ratio when exposed to waterlogging (**Table 1**). As proposed, an increased root/shoot ratio would increase absorbent root surface and further improve water and nutrient use to enhance tolerance of plants under stress conditions (Hebeisen et al., 1997; Lazzarotto et al., 2009). Besides, previous studies have reported resource allocation was a response of plant to water stress (Blanch et al., 1999; Reents et al., 2021). However, the allocation of photosynthetic carbon in the underground part is likely to change with soil physicochemical properties (Wang et al., 2019). For instance, soil nutrient shortages will increase the proportion of plant photosynthates to roots in wetlands (Cronin and Lodge, 2003). Further studies are warranted to explore the potential effects of soil physicochemical properties in shaping how plants respond to water stress.

### 4.2 Photosynthetic pigments

Our study agrees with previous studies suggesting that the contents of chlorophyll a and b showed a species-dependent response to water stress (Zaefyzadeh et al., 2009; Bhusal et al., 2020b). We observed that photosynthetic pigments in the *P. crymophila* significantly increased under water stress (**Table 2**). Previous studies demonstrated that drought stress and water deficit decreased (Din et al., 2011; Sperdouli and Moustakas, 2012; Chen et al., 2016; Meher et al., 2018; El-Beltagi et al., 2020) or increased (Balbaa et al., 2022) the chlorophyll content of leaves. In addition, waterlogging also exerts a negative effect on chlorophyll content and photosynthesis (Du et al., 2012; Bai et al., 2013; Barickman et al., 2019; Bhusal et al., 2020a). The decrease in chlorophyll content may be because drought or waterlogging induced the production of reactive oxygen species (ROS), such as O_2_ and H_2_O_2_, which led to lipid peroxidation and consequently chlorophyll destruction. We observed that moderate drought decreased chlorophyll a and b contents as well as carotenoid content. In previous studies, drought decreased chlorophyll a and b contents as well as carotenoid contents in green grams (Anosheh et al., 2012), and moderate drought also decreased chlorophyll a and b in *Salvia officinalis* (Caser et al., 2019).

### 4.3 MDA content

Earlier studies suggested that drought did not change (Loggini et al., 1999) or increased lipid peroxidation (Moreno-Galván et al., 2020), whereas waterlogging increased lipid peroxidation (Tan et al., 2008; Jumrani and Bhatia, 2019). In our study, we found that there were significant plant species effect and interactive effects of water regime and plant species on lipid peroxidation (**Figure 1**; **Table A2**), implying that plant tolerance to abiotic stress can be context dependent; the interspecies inherent difference in adoption tactics would change with living conditions. From the perspective of lipid peroxidation, *D. caespitosa* seems to be suitable for both waterlogging and water deficit conditions. The possible reason why the content of MDA remained fairly stable under water stresses may be related to either effective scavenging of free radicals by the antioxidant system or the prevention of free radical production (Tokarz et al., 2020).

### 4.4 Osmoprotective compounds

Our findings imply that the interspecies differences in the contents of soluble sugar, betaine, soluble protein and proline in plant shoots and roots changed greatly with their habitats (**Table A**; **Figure 2**). Specifically, we observed divergent effects of water stress on soluble sugar in plants (**Figure 2C**). Previous studies found that drought stress significantly increased the levels of sugars, betaines and proline (Chaves and Oliveira, 2004; Anosheh et al., 2012). Additionally, plants can also cope with water or osmotic stress by increasing the synthesis of osmoprotectants, such as proline (Sperdouli and Moustakas, 2012), an amino acid, exhibiting a dual function as an osmolyte compound and as an antioxidant when plants are exposed to various stresses (Hayat et al., 2012; Sperdouli and Moustakas, 2012; El-Beltagi et al., 2020). Studies have proposed that drought triggers modifications in proline metabolism that impair plant stress tolerance. Our findings are in agreement with studies suggesting that the proline content of plants under drought stress significantly increased compared with that of untreated plants (Hare et al., 1998; Anosheh et al., 2012; Sperdouli and Moustakas, 2012; Jayant and Sarangi, 2014; Jumrani and Bhatia, 2019; El-Beltagi et al., 2020).

## 5 Conclusions

In summary, we found significant effects of water stress on plant height, root length, total biomass, root/shoot ratio, and contents of chlorophyll a, chlorophyll b, chlorophyll (a+b), carotenoids, malondialdehyde and soluble sugar. We also observed apparent interspecies differences in plant height, root length, total biomass, root/shoot ratio, and contents of chlorophyll a, chlorophyll b, chlorophyll (a+b), carotenoids, malondialdehyde, soluble sugar, betaine, soluble protein and proline. Finally, we observed significant interactive effects of water stress and species identity on plant height, root length, total biomass, root/shoot ratio, and contents of chlorophyll a, chlorophyll b, chlorophyll (a+b), carotenoids, malondialdehyde, soluble sugar, betaine, soluble protein and proline. However, the interactive effects of water stress and plant species identity on some examined parameters changed with plant tissue. Our results yield important implications for our understanding of ecosystem resilience to water stresses as well as plant species distribution in the Qinghai-Tibetan Plateau. We argue that these findings could provide a fundamental basis for the identification of tolerant germplasm resources to restore the degraded grassland and wetlands under future intensive global climate change.

## Data availability statement

The original contributions presented in the study are included in the article/**Supplementary Material**. Further inquiries can be directed to the corresponding author.

## Author contributions

YSM designed the experiments. QL, HX and YGM collected the samples. QL and HY performed the laboratory work. QL, ZC and BY analyzed the data. BY contributed to manuscript revision. QL wrote the first version of the manuscript, which was then edited by all co-authors. All authors contributed to the article and approved the submitted version.
