# Supplementary material for: Morphology, photosynthetic physiology and biochemistry of nine herbaceous plants under water stress
Source: Front Plant Sci. 2023 Mar 30;14:1147208. doi: 10.3389/fpls.2023.1147208 (PMC10098446; doi:10.3389/fpls.2023.1147208)
Supplement: Supplementary file 1 [file Table_1.docx]

Table A1 Results of two-way ANOVAs examining the major and interactive effects of water regime and plant species identity on plant morphological characteristics

| Source of variation | df | Plant height | | Root length | | Total biomass | | Root/shoot ratio | |
| --- | --- | --- | --- | --- | --- | --- | --- | --- | --- |
|  |  | F | P | F | P | F | P | F | P |
| Water regime (W) | 2 | 6.319 | < 0.001 | 7.650 | < 0.001 | 9.758 | < 0.001 | 45.368 | < 0.001 |
| Species identity (S) | 8 | 4.921 | 0.011 | 3.132 | 0.052 | 9.748 | < 0.001 | 76.295 | < 0.001 |
| Interaction (W×S) | 16 | 2.921 | 0.002 | 1.225 | 0.280 | 9.468 | < 0.001 | 15.670 | < 0.001 |
