# Supplementary material for: Morphology, photosynthetic physiology and biochemistry of nine herbaceous plants under water stress
Source: Front Plant Sci. 2023 Mar 30;14:1147208. doi: 10.3389/fpls.2023.1147208 (PMC10098446; doi:10.3389/fpls.2023.1147208)
Supplement: Supplementary file 2 [file Table_2.docx]

Table A2 Results of two-way ANOVAs examining the major and interactive effects of water regime and plant species identity on malonaldehyde contents in shoots and root of nine selected plants

| Source of variation | df | Shoot | | Root | |
| --- | --- | --- | --- | --- | --- |
|  |  | F | P | F | P |
| Water regime (W) | 2 | 7.753 | < 0.001 | 20.435 | < 0.001 |
| Species identity (S) | 8 | 12.653 | < 0.001 | 3.438 | 0.039 |
| interaction (W×S) | 16 | 10.377 | < 0.001 | 1.575 | 0.108 |
