# Supplementary material for: Morphology, photosynthetic physiology and biochemistry of nine herbaceous plants under water stress
Source: Front Plant Sci. 2023 Mar 30;14:1147208. doi: 10.3389/fpls.2023.1147208 (PMC10098446; doi:10.3389/fpls.2023.1147208)
Supplement: Supplementary file 3 [file Table_3.docx]

Table A3 Results of two-way ANOVAs examining the major and interactive effects of water regime and plant species identity on osmoprotective compound content in shoots and roots of nine selected plants

| Part | Source of variation | df | Soluble protein | | Betaine | | Soluble sugar | | Proline | |
| --- | --- | --- | --- | --- | --- | --- | --- | --- | --- | --- |
|  |  |  | F | P | F | P | F | P | F | P |
| Shoot | Water regime (W) | 2 | 163.927 | < 0.001 | 38.527 | < 0.001 | 37.019 | < 0.001 | 97.654 | < 0.001 |
|  | Species identity (S) | 8 | 1.609 | 0.210 | 0.549 | 0.581 | 74.013 | < 0.001 | 77.911 | < 0.001 |
|  | Interaction (W×S) | 16 | 12.392 | < 0.001 | 8.447 | < 0.001 | 23.291 | < 0.001 | 34.337 | < 0.001 |
| Root | Water regime (W) | 2 | 164.887 | < 0.001 | 83.012 | < 0.001 | 252.512 | < 0.001 | 129.849 | < 0.001 |
|  | Species identity (S) | 8 | 20.603 | < 0.001 | 4.480 | 0.016 | 181.323 | < 0.001 | 59.687 | < 0.001 |
|  | Interaction (W×S) | 16 | 10.951 | < 0.001 | 13.969 | < 0.001 | 92.417 | < 0.001 | 36.275 | < 0.001 |
